# Evaluation Expression of miR-146a and miR-155 in Non-Small-Cell Lung Cancer Patients

**DOI:** 10.3389/fonc.2021.715677

**Published:** 2021-11-01

**Authors:** Neda K. Dezfuli, Shamila D. Alipoor, Neda Dalil Roofchayee, Sharareh Seyfi, Babak Salimi, Ian M. Adcock, Esmaeil Mortaz

**Affiliations:** ^1^ Clinical Tuberculosis and Epidemiology Research Center, National Research Institute of Tuberculosis and Lung Diseases (NRITLD), Shahid Beheshti University of Medical Sciences, Tehran, Iran; ^2^ Department of Immunology and Laboratory Sciences, School of Allied Medical Sciences, Dezful University of Medical Sciences, Dezful, Iran; ^3^ Molecular Medicine Department, Institute of Medical Biotechnology, National Institute of Genetic Engineering and Biotechnology, Tehran, Iran; ^4^ Department of Immunology, School of Medicine, Shahid Beheshti University of Medical Sciences, Tehran, Iran; ^5^ Chronic Respiratory Diseases Research Center, National Research Institute of Tuberculosis and Lung Diseases (NRITLD), Shahid Beheshti University of Medical Sciences, Tehran, Iran; ^6^ Airways Disease Section, National Heart and Lung Institute, Imperial College London, London, United Kingdom

**Keywords:** cytokines, immune system, NSCLC, miR-155, miR-146a

## Abstract

**Background:**

Non−small-cell lung cancer (NSCLC) is the major type of lung cancer. MicroRNAs (miRNAs) are novel markers and targets in cancer therapy and can act as both tumor suppressors and oncogenes and affect immune function. The aim of this study was to investigate the expression of miR146a and miR155 in linked to blood immune cell phenotypes and serum cytokines in NSCLC patients.

**Methods:**

Thirty-three NSCLC patients and 30 healthy subjects were enrolled in this study. The allele frequencies of potential DNA polymorphisms were studied using polymerase chain reaction (PCR)–restriction fragment length polymorphism (PCR-RFLP) analysis in peripheral blood samples. Quantitative reverse transcription PCR (qRT-PCR) was used to measure the expression of miR-146a and miR-155 in peripheral blood mononuclear cells (PBMCs). Serum cytokine (IL-1β, IL-6, TNF-α, TGF-β, IL-4, IFN-γ) levels were determined by ELISA. The frequency of circulating CD3+CTLA-4+ and CD4+CD25+FOXP3+ (T regulatory cells/Treg) expression was measured by flow cytometry.

**Results:**

miR-146a was significantly downregulated in PBMC of NSCLC patients (P ≤ 0.001). Moreover, IL-6 and TGF-β levels were elevated in NSCLC patients (P ≤ 0.001, P ≤ 0.018, respectively). CD3+ CTLA-4+ and Treg cells frequencies were higher in patients than in control subjects (P ≤ 0.0001, P ≤ 0.0001, respectively). There was a positive correlation between miR-155 and IL-1β levels (r=0.567, p ≤ 0.001) and a negative correlation between miR-146a and TGF-β levels (r=-0.376, P ≤ 0.031) in NSCLC patients. No significant differences were found in the relative expression of miR-146a and miR-155, cytokine levels or immune cell numbers according to miR-146a and miR-155 (GG/GC/CC, TT/AT/AA) genotypes. However, there was a positive correlation between miR-146a and IL-1β levels (r=0.74, P ≤ 0.009) in GG subjects and a positive correlation between miR-146a expression and CD3+CTLA4+ cell frequency (r=0.79, P ≤ 0.01) in CC genotyped subjects. Conversely, a negative correlation between miR-146a expression and Treg cell frequency (r=−0.87, P ≤ 0.05) was observed with the GG genotype. A positive correlation between miR-155 and IL-1β expression (r=0.58, p ≤ 0.009) in the TT genotype and between miR-155 expression and CD3+CTLA-4 cell frequency (r=0.75, P ≤ 0.01) was observed in the AT genotype.

**Conclusions:**

The current data suggest that the miR-146a expression in PBMC and serum TGF-β and IL-1β levels may act as blood markers in NSCLC patients. Further study is needed to elucidate the link between immune cells and serum miR146 at early disease stages.

## Introduction

Lung cancer is one of the leading causes of cancer mortality worldwide, accounting for more than 1.4 million deaths per year (1). The two major types of lung cancer are non-small-cell lung cancer (NSCLC) (responsible for 85% of all lung cancers) and small-cell lung cancer (about 15% of all lung cancer) (2). Despite improvements in early diagnosis and new therapeutic strategies, 5-year survival rates remain at 10–20% (1). The poor prognosis is due to various factors including diagnosis at advanced disease stages, tumor heterogeneity, and relatively limited understanding of lung cancer biology (3). In the last decade, immune checkpoint antibodies against markers of exhausted T cells such as PD-1 (programmed death protein)/PD-L1 (programmed death protein ligand) and CTLA-4 (cytotoxic T-cell lymphocyte antigen 4) have been successful in treating multiple solid tumor malignancies including lung cancer (4, 5).

Surgical resection is the most common treatment for early-stage tumors and is used in combination with chemotherapeutic agents for advanced lung cancer patients. In addition, chemotherapy treatment is required for metastatic disease (6–8). Recent studies have shown large increases in the survival of lung cancer patients since the introduction of targeted and immune-based therapies.

Early detection of lung cancer is critical (9), and the immune system is a key player in the development and progression of cancers (10). Tumors often arise at the sites of chronic inflammation linked to the presence of distinct immune cells in the tumor milieu. The immune system plays a critical role in the progression of cancer by releasing pro- or antitumorigenic factors (11). Non-coding microRNAs (mRNAs) are novel mediators of the immune response associated with inflammation and cancer development (12). miRNA expression is important in tumor cell function, and they indicate disease progression and response to therapy (13). Dysregulated miRNA production has been reported in several chronic inflammatory diseases (14) where they modulate immune responses. In particular, miR-146 (146a and 146b) and miR-155 have been reported as being essential in regulating the immune system (15).

Previously, we have shown that miR-146a rs2910164 and miR-155 rs767649 polymorphisms may act as genetic risk factors for the susceptibility to Iranian NSCLC patients (16). Studies show an anti-inflammatory function of miR-146a, whereas miR-155 has an inflammatory function (15, 17, 18). Thus, understanding the pattern expression of these miRNAs could be useful in following cancer pathogenesis and progression (19). We hypothesize that relative levels of peripheral blood miR-146a and miR-155 expression may be used to diagnose NSCLC. We, therefore, evaluated miR-146a and miR-155 expression in PBMC and correlated this with blood Treg, CD3+CTLA-4+ cell, and serum cytokine levels in NSCLC patients.

## Material and Methods

### Study Participants

Thirty-three patients with newly diagnosed NSCLC (57.9 ± 9.5 years old, mean ± SD) were recruited at the Masih Daneshvari Hospital (Tehran, Iran) between April 2017 and September 2019. Histology and clinical parameters confirmed the presence of lung cancer, and patients were not on any treatment and had no history of other cancers or inflammatory diseases. Age- and sex-matched controls (n=30) were also recruited following a general health check and a negative history of cancer and inflammatory diseases (**Table 1**). The Ethics Committee of Shahid Beheshti University of Medical Sciences approved the study, and all subjects gave written informed consent (Ethics committee approval number: IR.SBMU.MSP.REC.1397.525).

**Table 1:** Demographic information of participants.

Parameters	Lung cancer, n = 33	Control, n = 30
**Age (Years, Mean ± SD)**	57.9 ± 9.5	53.3 ± 7.7
**Gender (n, %)**		
**Female**	9	9
**Male**	24	21
**Smoking status (n, %)**		
**Smoker**	16	13
**Non-smoker**	17	17
**Histological subtype (n, %)**		
**ADC**	27	
**LCC**	1	
**SCC**	5	
**Stage (n, %)**		
**I**	1	
**II**	3	
**III**	8	
**IV**	21	
**MiR-146a Genotype**		
**GG**	11	10
**GC**	14	14
**CC**	8	6
**MiR-155 Genotype**		
**TT**	19	17
**AT**	10	9
**AA**	4	4

ADC, adenocarcinoma; LCC, large-cell carcinoma; SCC, squamous cell carcinoma.

### Sampling Procedure

Ten ml whole blood samples from healthy control and patients groups was collected into separate tubes containing blood clot activating gel for obtaining serum (cytokine assay), heparin tubes for PBMC isolation, and EDTA tubes for flow cytometry.

### Genotyping of miR-146a and miR-155 for Possible SNP

Genomic DNA was isolated from peripheral blood cells using a DNA extraction kit (High Pure PCR template preparation kit, Roche, Germany, cat. No.11796828001) according to the manufacturer’s instructions. The concentration and quality of DNA was measured by Nanodrop 2000 (Thermo Fisher Scientific, USA). Specific SNPs (Rs2910164 and Rs767649) were genotyped using PCR-Restriction Fragment Length Polymorphism (RFLP). PCR reactions were performed using super PCR master mix (YEKTA TAJHIZ AZMA, Tehran, Iran, Cat No: YT1553-YT1554) using a Thermal Cycler (Bio-Rad, CA, USA). The purity of the samples was assessed using 260/280 nm and 260/230 nm ratios, and the concentrations of isolated DNA from healthy subjects and patients are presented in **Supplementary Tables 1, 2**. The primer sequences for each PCR reaction are shown in **Table 2**. The cycle parameters for the PCR analysis were as follows: initial denaturation at 95°C for 5 min, 35 cycles of denaturation at 94°C for 30 s, annealing at 58°C for 1 min and extension at 72°C for 1 min, and a final extension at 72°C for 10 min. To identify the miR-146 C/G polymorphism, the PCR product was digested with the restriction enzyme mnlI (Thermo Fisher, USA, Cat No. ER1071) at 37°C for 4 h. The PCR product (miR-155 T/A polymorphism) was incubated at 37°C for 4 h with the restriction enzyme TSP45I (Thermo Fisher, USA, Cat No. ER1511) and the digestion products detected by 3% agarose gel electrophoresis.

**Table 2:** PCR primer sequences used and expected fragment sizes.

Polymorphism	Primer sequence	Restriction enzyme	Product size (bp)
**Rs2910164**	F: 5’-AGAACTGAATTCCATGGGTTG-3’	mnlI	Uncut product: 248
	R: 5’-TGCTTAGCATAGAATTCAAGTC-3’		G Allele: 171 + 77
			C Allele: 171 + 45+32
**Rs767649**	F: 5’-CCT GTA TGA CAA GGT TGT GTT TG-3’	TSP451	Uncut product: 294
	R: 5’-GCT GGC ATA CTA TTC TAC CCA TAA-3’		A Allele: 252 + 42
			T Allele: 158 + 94+42

### PBMCs Isolation

Whole blood (5 ml) was collected in heparin-containing tubes, and peripheral blood mononuclear cells (PBMCs) were isolated using density gradient centrifugation. Briefly, the blood was diluted with equal volume of PBS buffer and then slowly added to 5 ml lymphocyte separation medium (Ficoll Paque, BAG Health Care GmbH, Germany, Cat No: 70125). After centrifuging at 278 × g at room temperature (RT) for 30 min, the supernatant was removed and the cell pellet washed with cold PBS. After one more wash and centrifugation, the supernatant was removed and 1 ml TRIzol (Invitrogen, CA, USA) was added to the cell pellet and stored at −80°C for isolation of RNA as described below.

### RNA Isolation and cDNA Synthesis

Total RNA was extracted from isolated PBMCs as described earlier (20). Briefly, cells containing TRIzol were treated by chloroform (Merck, Germany), and after isopropanol (Merck, Germany) sedimentation and ethanol washing, total RNA was diluted in sterile DEPC-treated water. The concentration and purity of RNA was determined by Nanodrop 2000 spectrophotometer (**Supplementary Tables 1, 2**). Extracted RNA was reverse transcribed using the miRCURY LNA Universal RT microRNA cDNA Synthesis Kit (miRCURY LNA RT Kit-QIAGEN, MD, USA) according to the manufacturer’s instructions.

### Quantitative RT-PCR Analysis

miR146a and miR-155 were detected by real-time PCR assays by using the SYBR Green Master Mix kit (QIAGEN, MD, USA). miRNA primers were purchased from QIAGEN (has-miR-146A-5p, Cat. No YP00204688; has-miR-155-5p, Cat no. YP00204308), and quantitative PCR was performed using Real-time PCR (Roche, Manheim, Germany). The real-time PCR program included the following steps: an initial denaturation step at 95°C for 10 min; 45 amplification cycles that consisted of a denaturation step (10 s at 95°C) and an annealing step (60 s at 60°C). Expression levels of miRNAs were normalized to the level of miR-16 (QIAGEN, MD, USA) as a control miRNA using the 2^– ΔΔCt^ method.

### Flow Cytometry Assay

Two ml whole blood containing EDTA was collected from participants. To determine the immunophenotyping of T cells, surface staining of CD4 and CD25 markers was performed using mouse antihuman CD25-FITC (Biolegend, San Diego, CA, USA) and CD4-PE (Immunostep, Salamanca, Spain) for 30 min at 4°C. Cells were then washed and incubated with fixation and permeabilization solution buffer (BD Biosciences, San Diego, CA, USA) for 15 min at 4°C. Subsequently, cells were washed with cold PBS and intracellular staining performed using a FOXP3-APC antibody (eBioscience, CA, USA) for 30 min in 4°C. Isotype-matched antibodies were used as controls for all the samples. Separate cells were incubated with CTLA-4-PE (Biolegend, San Diego, CA, USA) and CD3-APC (Biolegend, San Diego, CA, USA), for 30 min at 4°C with isotype-matched antibodies used as controls. Ten thousand events were evaluated by FACS Calibur (BD Biosciences). Data were processed using Flow Jo software version 8.

### Cytokine Analysis

Three ml whole blood in tubes without anticoagulants was harvested and after isolation of serum stored at −80°C. The levels of cytokines including IL-1β (R&D, Minneapolis, USA), IL-6 (R&D, Minneapolis, USA), TNF-α (R&D), IL-4 (Invitrogen, Vienna, Austria), IFN-γ (Invitrogen), and TGF-β1 (eBioscience, CA, USA) were measured in the serum of all participants according to the manufacturer’s instructions. The optical density was read by an ELISA plate-reader at a wavelength of 450 nm with a reference wavelength of 545 nm. All the assays were performed in duplicate on the same plate to be able to compare the groups.

### Statistical Analysis

Results were presented as the mean ± standard deviation (SD). Comparisons between two groups were analyzed using a Student’s *t*-test for the variables with a normal distribution and by the non-parametric Mann-Whitney U test for non-normally distributed data. Differences among multiple groups were compared using one-way analysis of variance (ANOVA) followed by a *post-hoc* Dunnett’s test. Receiver operating characteristic curve (ROC) analysis was applied to evaluate the potential of miRNA levels as diagnostic markers. Pearson correlation analysis was applied to measure the linear correlation between two sets of data. All statistical tests were carried out using SPSS-25 software (SPSS, Inc.). Graph Pad Prism software was used for drawing graphs. A P value ≤0.05 was considered statistically significant.

## Results

### Genotyping

Genotyping of patients and controls was performed by PCR-RFLP. The number of patients possessing miR-146a (GG/GC/CC) and miR-155 (TT/AT/AA) genotypes is shown together with their demographics in **Table 1**.

### Expression of miR-146a and miR-155 in PBMCs of NSCLC Patients

**Figure 1:**
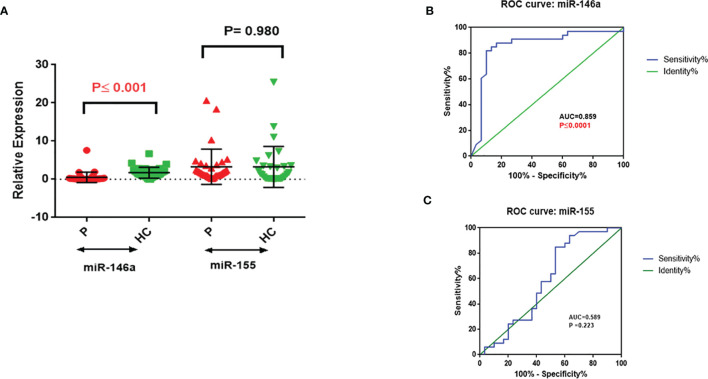
Relative expression of miR-146a and miR-155 in PBMC with ROC correlation in NSCLC and healthy control. **(A)** miR-146a and miR-155 relative expression; miR-146a significantly downregulated in NSCLC PBMCs. **(B)** miR-146a and **(C)** miR-155 expression level ROC curve; miR-146a identification of NSCLC patients from healthy controls.

miR-146a was significantly downregulated in PBMCs from NSCLC patients (P ≤ 0.001, **Figure 1A**), whereas miR-155 expression was not significantly different between NSCLC patients and the healthy control group (**Figure 1A**). ROC analysis of miR146 expression gave an AUC=0.859 (P ≤ 0.0001, Sensitivity: 81.82%, Specificity: 90%), whereas the criterion was >0.405 in NSCLC patients (**Figure 1B**). No significant differences were seen with the ROC analysis of miR-155 as a predictor of NSCLC (AUC=0.589, P=0.223) (**Figure 1C**).

### Serum Cytokines

**Figure 2:**
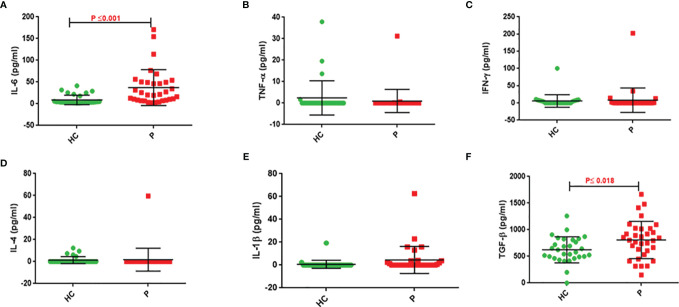
Serum cytokine levels of patients compared to control group. Serum IL-6 **(A)**, TNF-α **(B)**, IFN-γ **(C)**, IL-4 **(D)**, IL-1β **(E)**, and TGF-β **(F)** concentrations in serum of NSCLC patients and healthy subjects. Serum levels of IL-6 and TGF-β in NSCLC patients are significantly higher than that of controls (P ≤0.001 and 0 ≤ 0.018, respectively).

IL-6 and TGF-β levels in NSCLC patients were significantly higher than in healthy controls (P ≤ 0.001 and P ≤ 0.018, respectively). There were no differences in TNF-α, IL-1β, IFN-γ, and IL-4 levels between control subjects and NSCLC patients (**Table 3** and **Figure 2**).

**Table 3:** Cytokine results in patients and control groups.

Cytokine	Groups				P value (2-tailed)
	Patients (33)		Controls (30)		
	Mean (SD) pg/ml	Range (Min–Max) pg/ml	Mean (SD) pg/ml	Range (Min–Max) pg/ml	
**IL-1β**	4.5 (11.8)	0.01–19.3	0.73 (3.5)	0.01–62.7	0.089
**IL-6**	37.2 (41.2)	2.1–170.6	8.9 (11.0)	1.5–41.2	0.001
**TNF-α**	0.97 (5.4)	0.01–31.2	2.4 (7.9)	0.01–37.9	0.395
**IFN-γ**	8.1 (35.5)	0.01–202.4	5.9 (18.1)	0.01–100.3	0.759
**IL-4**	1.8 (10.3)	0.01–59.7	1.3 (3.2)	0.01–12.3	0.809
**TGF-β**	809.6 (349.9)	150.4–1665.7	623.7 (243.8)	0.00–1258.7	0.018

Comparisons between the groups were performed using Student’s t-test for the variables with a normal distribution and non-parametric Mann-Whitney U test for non-normally distributed data.

The values in red font are significant.

### Immunophenotyping of Treg and CD3+CTLA-4+ Lymphocytes

**Figure 3:**
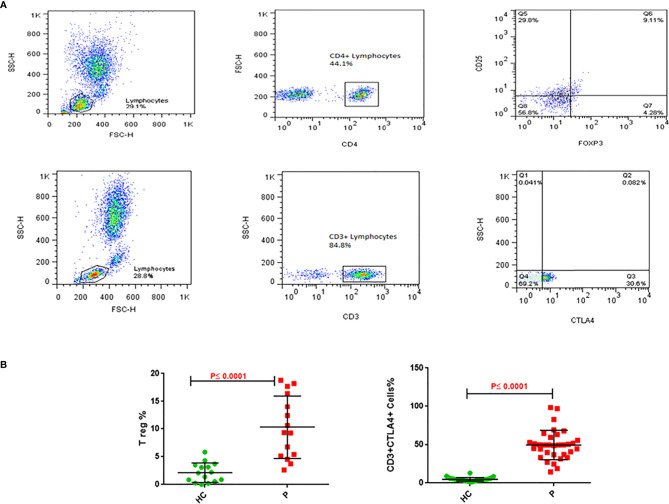
Treg and CD3/CTLA-4 cells in blood of NSCLC patients. (A) Upper panel: Gating strategy for Treg cells frequency; analysis of a blood sample for CD25, as well as FoxP3 expression in the CD4+ lymphocyte gate. **(A)** Lower panel: Gating strategy for CD3+CTLA4+ cells percentage. Analysis of a blood sample for CTLA4 expression in the CD3+ lymphocyte gate. **(B)** Flow cytometry analysis of frequencies Treg and CD3/CTLA-4 in blood cells stained with related antibodies of NSCLC and healthy control subjects, which was calculated statistically and plotted to the graph (P ≤ 0.0001). The dots indicate the means, and the error bars indicate the standard deviations of all patients and healthy control measurements.

The immunophenotyping gating strategy for Treg (CD4+ CD25+ FOXP3+) and CD3+ CTLA-4+ T cells is depicted in representative samples from participants in **Figure 3A** (Upper panel for Treg cells and lower panel for CD3+CTLA-4+ cells). The frequency of Treg cells in PBMCs from NSCLC patients was five-fold greater than in healthy controls (10.3 *vs* 2.1%, P≤0.0001, **Figure 3B** left panel), whereas that for CD3+CTLA4+bearing lymphocytes was 10-fold greater in NSCLC patients compared to healthy controls (49.3 *vs* 4.8%, P≤0.0001, **Figure 3B** right panel) (**Table 4**).

**Table 4:** Flow cytometry results of Treg and CD3/CTLA4 in patients and control.

	Groups	N	Mean (Min–Max)	SD	P value (2-tailed)
**CD4+CD25+Foxp3+ lymphocytes**	Patients	15	10.3% (2.6–18.8%)	5.6	≤0.0001
	Control	15	2.1% (0.00–5.8%)	1.7	
**CD3+CTLA4+ lymphocytes**	Patients	33	49.3% (14.5–98.2%)	19.1	≤0.0001
	Control	30	4.8% (1.1–12.9%)	2.4	

Comparisons between the groups were performed using Student’s t-test for the variables with a normal distribution and non-parametric Mann-Whitney U test for non-normally distributed data. N; numbers.

The values in red font are significant.

### Correlation Analysis

**Figure 4:**
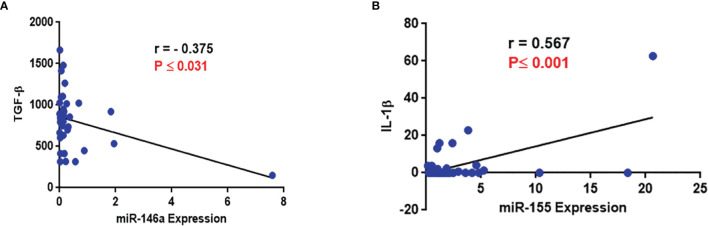
Evaluation of correlation of miR-146a and miR-155 with cytokines **(A)** Correlation between miR-146a and TGF-β level; a negative correlation between miR-146a and TGF-β level was observed. **(B)** Correlation between miR-155 and IL-1β level; a positive correlation between miR-155 and IL-1β level was observed.

No correlation was seen between any of the variables analyzed with the type and stages of NSCLC except for significantly reduced IL-6 levels among patients with Stage III disease (**Table 5**). There was a negative correlation between miR-146a and TGF-β expression (r=−0.376, P ≤ 0.031, **Table 6** and **Figure 4A**) and a positive correlation between miR-155 and IL-1β levels (r=0.567, p ≤ 0.001, **Table 6** and **Figure 4B**).

**Table 5:** Data analysis on correlation of NSCLC patients with type and stages of diseases with studied parameters.

Parameters	NSCLC type	N	Mean ± SD	P value ADC&SCC	NSCLC Stage	N	Mean ± SD	P value II&III	P value II&IV	P value III&IV
**miR-146a expression**	ADC	27	0.29 ± 0.49	0.33	II	3	0.25 ± 0.37	0.50	0.95	0.27
					III	8	1.33 ± 2.59			
	SCC	5	1.69 ± 3.3		IV	21	0.23 ± 0.43			
**miR-155 expression**	ADC	27	3.56 ± 5.06	0.49	II	3	2.07 ± 1.36	0.98	0.64	0.44
					III	8	2.04 ± 1.67			
	SCC	5	1.95 ± 1.6		IV	21	3.58 ± 5.46			
**IL-1β** (pg/ml)	ADC	27	4.59 ± 12.5	0.94	II	3	0.01 ± 0.0	0.50	0.48	0.63
					III	8	3.2 ± 7.96			
	SCC	5	4.99 ± 10		IV	21	5.84 ± 14.0			
**IL-6** (pg/ml)	ADC	27	37.2 ± 41.8	0.80	II	3	71.6 ± 37.1	0.03	0.25	0.41
					III	8	24.5 ± 23.7			
	SCC	5	42.4 ± 45.3		IV	21	38.6 ± 45.8			
**TNF-α** (pg/ml)	ADC	27	1.17 ± 6.0	0.70	II	3	0.01 ± 0.0	1.0	0.70	0.53
					III	8	0.01 ± 0.0			
	SCC	5	0.12 ± 0.25		IV	21	1.53 ± 6.80			
**IFN-γ** (pg/ml)	ADC	27	9.91 ± 39.1	0.58	II	3	0.01 ± 0.0	1.0	0.62	0.42
					III	8	0.01 ± 0.0			
	SCC	5	0.01 ± 0.01		IV	21	12.7 ± 44.2			
**IL-4** (pg/ml)	ADC	27	2.22 ± 11.4	0.67	II	3	0.01 ± 0.0	1.0	0.71	0.54
					III	8	0.01 ± 0.0			
	SCC	5	0.01 ± 0.0		IV	21	2.85 ± 13.0			
**TGF-β** (pg/ml)	ADC	27	839.4 ± 345.3	0.24	II	3	819.2 ± 185.3	0.94	0.93	0.99
					III	8	800.1 ± 478.0			
	SCC	5	637.0 ± 400.7		IV	21	802.0 ± 332.1			
**Treg Cells (%)**	ADC	12	10.42 ± 6.0	0.89	II	3	9.08 ± 5.83	0.38	0.90	0.26
					III	3	13.98 ± 6.37			
	SCC	3	9.90 ± 4.4		IV	9	9.51 ± 5.53			
**CD3+CTLA4+ Cells (%)**	ADC	27	47.4 ± 18.4	0.12	II	3	67.1 ± 26.9	0.36	0.07	0.33
					III	8	53.1 ± 19.5			
	SCC	5	62.0 ± 21.0		IV	21	45.2 ± 17.5			

Comparisons between NSCLC types and stages were performed using Student’s t-test for the variables with a normal distribution and non-parametric Mann-Whitney U test for non-normally distributed data.

The values in red font are significant.

**Table 6:** Association between miR-146a and miR-155 expression with cytokines and Treg and CD3/CTALA-4 in patients.

Parameters	N	MiR-146a Expression		MiR-155 Expression	
		Pearson correlation (r)	P value (2-tailed)	Pearson correlation (r)	P value (2-tailed)
**IL-1β** (pg/ml)	33	0.224	0.209	0.567	0.001
**IL-6** (pg/ml)	33	−0.243	0.173	−0.150	0.403
**TNF-α** (pg/ml)	33	−0.055	0.761	−0.016	0.931
**IFN–γ** (pg/ml)	33	−0.085	0.639	−0.089	0.624
**IL-4** (pg/ml)	33	0.191	0.288	−0.078	0.666
**TGF-β** (pg/ml)	33	−0.376	0.031	0.030	0.869
**CD4+CD25+Foxp3+ lymphocytes (%)**	15	−0.296	0.283	0.415	0.124
**CD3+CTLA4+ Lymphocytes (%)**	33	−0.056	0.757	0.024	0.894

Data are analyzed using linear regression, and r values are from Pearson’s correlation coefficient test.

The values in red font are significant.

There was no significant effect of the Rs2910164 genotypes (GG/GC/CC) on miR-146a expression or on the levels of cytokines and Treg and CD3+CTLA-4+ cells in NSCLC patients and control subjects (**Table 7**). Furthermore, there was no effect of the Rs767649 genotypes (TT/AT/AA) on miR-155 expression levels or on the levels of cytokines, Treg, and CD3/CTLA-4 cells in the patient and control groups (**Table 8**).

**Table 7:** Effect of the Rs2910164 genotypes on miR-146a expression, cytokines, and Treg, CD3+CTLA-4+ cells.

Parameters	Genotype	Patients				Controls			
		N	Mean	SD	P	N	Mean	SD	P
**miR-146a expression**	GG	11	1.22	2.22	0.092	10	2.45	1.90	0.081
	GC	14	0.12	0.14		14	1.62	1.05	
	CC	8	0.16	0.22		6	0.87	0.46	
**IL-1β** (pg/ml)	GG	11	3.59	7.97	0.593	10	2.17	6.07	0.29
	GC	14	3.05	5.11		14	0.01	0.00	
	CC	8	8.32	22.02		6	0.01	0.00	
**IL-6** (pg/ml)	GG	11	16.67	21.93	0.067	10	3.18	1.04	0.061
	GC	14	54.74	53.32		14	9.89	12.02	
	CC	8	34.93	23.26		6	16.18	13.99	
**TNF-α** (pg/ml)	GG	11	2.84	9.41	0.388	10	0.01	0.00	0.207
	GC	14	0.02	0.03		14	5.20	11.19	
	CC	8	0.08	0.19		6	0.01	0.00	
**IFN-γ** (pg/ml)	GG	11	1.25	3.91	0.401	10	2.10	3.46	0.650
	GC	14	18.04	53.93		14	9.16	26.47	
	CC	8	0.28	0.69		6	4.71	4.11	
**IL-4** (pg/ml)	GG	11	5.44	18.00	0.380	10	1.81	2.97	0.596
	GC	14	0.01	0.00		14	0.68	2.53	
	CC	8	0.01	0.00		6	2.06	5.03	
**TGF-β** (pg/ml)	GG	11	715.81	340.98	0.112	10	558.30	135.77	0.218
	GC	14	756.33	289.71		14	707.10	251.46	
	CC	8	103.19	402.48		6	538.13	332.41	
**CD4+CD25+Foxp3+ lymphocytes (%)**	GG	5	11.42	6.47	0.845	5	1.34	1.45	0.324
	GC	5	9.20	6.07		5	3.03	2.33	
	CC	5	10.34	5.42		5	2.01	1.11	
**CD3+CTLA4+ Lymphocytes (%)**	GG	11	44.23	13.44	0.548	10	4.80	3.24	0.726
	GC	14	50.95	21.69		14	1.83	0.48	
	CC	8	53.48	21.72		6	2.37	0.96	

Differences among three groups were compared using one-way analysis of variance (ANOVA) followed by post-hoc Dunnett’s test.

**Table 8:** Effect of the Rs767649 genotypes on miR-155 expression, cytokines, and Treg, CD3+CTLA-4+ cells.

Parameters	Genotype	Patients				Controls			
		N	Mean	SD	P	N	Mean	SD	P
**miR-155 Expression**	TT	19	4.69	5.75	0.124	17	5.11	6.53	0.084
	AT	10	1.36	0.46		9	1.10	1.14	
	AA	4	1.35	0.69		4	0.14	0.06	
**IL-1β** (pg/ml)	TT	19	6.18	14.98	0.597	17	0.01	0.00	0.239
	AT	10	3.15	6.08		9	2.41	6.39	
	AA	4	0.01	0.00		4	0.01	0.00	
**IL-6** (pg/ml)	TT	19	37.76	40.21	0.810	17	8.14	9.62	0.399
	AT	10	41.18	50.96		9	12.60	15.02	
	AA	4	24.97	20.77		4	3.92	1.54	
**TNF-α** (pg/ml)	TT	19	1.66	7.16	0.714	17	4.28	10.29	0.356
	AT	10	0.01	0.00		9	0.01	0.00	
	AA	4	0.15	0.28		4	0.01	0.00	
**IFN-γ** (pg/ml)	TT	19	1.60	4.30	0.257	17	8.93	23.87	0.596
	AT	10	23.73	63.71		9	1.63	3.37	
	AA	4	0.22	0.42		4	2.73	3.39	
**IL-4** (pg/ml)	TT	19	0.01	0.00	0.327	17	1.43	3.46	0.673
	AT	10	5.98	18.88		9	1.74	3.54	
	AA	4	0.01	0.00		4	0.01	0.00	
**TGF-β** (pg/ml)	TT	19	747.51	362.40	0.342	17	674.65	297.33	0.437
	AT	10	946.76	346.64		9	552.42	118.36	
	AA	4	761.86	260.50		4	567.59	178.42	
**CD4+CD25+Foxp3+ lymphocytes (%)**	TT	6	12.30	4.78	0.470	6	3.10	1.82	0.175
	AT	6	8.14	6.26		6	1.75	1.62	
	AA	3	10.71	6.40		3	0.94	0.99	
**CD3+CTLA4+ Lymphocytes (%)**	TT	19	53.06	15.39	0.432	17	4.90	1.81	0.447
	AT	10	43.69	26.72		9	5.23	3.53	
	AA	4	45.67	10.96		4	3.39	1.67	

Difference among three groups was compared using one−way analysis of variance (ANOVA) followed by post-hoc Dunnett’s test.

In contrast, there was a positive correlation between miR-146a and IL-1β levels (r=0.74, P ≤ 0.009) with the GG genotype and with the frequency of CD3+CTLA4+ cells (r=0.79, P ≤ 0.01) in CC genotype. Finally, there was a negative correlation between miR-146a expression and Treg cell frequency (r=−0.87, P ≤ 0.05) in patients with the GG genotype; a positive correlation between miR-155 and IL-1β (r=0.58, p ≤ 0.009) in the TT genotype and with the frequency of CD3+CTLA4+ cells (r=0.75, P ≤ 0.01) in patients with the AT genotype (**Table 9**).

**Table 9:** Correlation between miR-146a and miR-155 expression with the level of cytokines, Treg, and CD3+CTLA-4+ cells in patients based on genotypes.

Parameter	Genotype	N	miR-146a Expression		Genotype	N	miR-155 Expression	
			Pearson correlation(r)	P value(2-tailed)			Pearson correlation(r)	P value(2-tailed)
**IL-1β** (pg/ml)	GG	11	0.74	0.009	TT	19	0.58	0.009
	GC	14	−0.30	0.29	AT	10	−0.25	0.48
	CC	8	−0.08	0.84	AA	4	−0.22	0.77
**IL-6** (pg/ml)	GG	11	−0.34	0.30	TT	19	−0.21	0.38
	GC	14	−0.27	0.35	AT	10	−0.25	0.48
	CC	8	0.22	0.59	AA	4	−0.54	0.45
**TNF-α** (pg/ml)	GG	11	−0.16	0.62	TT	19	−0.7	0.76
	GC	14	−0.21	0.46	AT	10	*	*
	CC	8	−0.29	0.47	AA	4	−0.89	0.10
**IFN-γ** (pg/ml)	GG	11	−0.17	0.60	TT	19	0.01	0.96
	GC	14	−0.28	0.31	AT	10	−0.22	0.53
	CC	8	−0.09	0.82	AA	4	0.68	0.31
**IL-4** (pg/ml)	GG	11	0.10	0.75	TT	19	*	*
	GC	14	*	*	AT	10	−0.07	0.83
	CC	8	*	*	AA	4	*	*
**TGF-β** (pg/ml)	GG	11	−0.57	0.06	TT	19	0.15	0.52
	GC	14	−0.26	0.35	AT	10	−0.16	0.64
	CC	8	−0.21	0.60	AA	4	−0.31	0.68
**Treg Cells (%)**	GG	5	−0.87	0.05	TT	6	0.56	0.24
	GC	5	0.61	0.26	AT	6	0.21	0.68
	CC	5	−0.02	0.96	AA	3	0.60	0.58
**CD3+CTLA4+ Cells (%)**	GG	11	−0.05	0.86	TT	19	−0.15	0.53
	GC	14	0.09	0.73	AT	10	0.75	0.01
	CC	8	0.79	0.01	AA	4	−0.54	0.45

Data are analyzed using linear regression, and r values are from Pearson’s correlation coefficient test.

*Cannot be computed because at least one of the variables is constant.

The values in red font are significant.

## Discussion

We found that miR-146a expression in PBMCs is downregulated in NSCLC patients compared to healthy control subjects. In contrast, serum IL-6 and TGF-β levels as well as CD3+ CTLA4+ and Treg cell frequencies in blood were elevated in NSCLC patients. In addition, the expression of miR-146a is negatively associated with a high serum level of TGF-β. No differences in the expression of miR-155 between patients and controls was found, although miR-155 expression positively associated with higher serum levels of IL-1β. In addition, there was a positive correlation between miR-146a expression and IL-1β levels in patients with a GG genotype and with CD3+CTLA4+ cells frequency in the CC genotype. There was a negative correlation between miR-146a and Treg cells frequency in patients with a GG genotype. Finally, there was a positive correlation between miR-155 expression and IL-1β in the TT genotype and CD3+CTLA4+ cells frequency in the AT genotype.

To our knowledge this is the first study showing miR-146a and miR-155 expression levels in PBMC of NSCLC patients. The importance of miR-146a downregulation and higher levels of serum cytokines, Treg, and CTLA4+ cells in relation to the biological and clinical aspects of NSCLC needs to be further examined. miR-146a is involved in the development of various cancers and in suppressing inflammation through the modulation of the innate immune response (21, 22). Larger, multicentered studies should investigate whether this correlation between TGF-β and IL-1β with the expression of miR-146a and miR-155 is validated and has a clinical impact in the pathophysiology of NSCLC patients.

miR-146a and miR-146b are members of the miR-146 family, which are found on chromosomes 5 and 10. miR-146a and miR-146b have similar structure but a different mature sequence (23). miR-146a has an important role in cell signaling and regulation of toll like receptor (TLR) pathways (24). Knockout of miR-146a leads to excessive production of inflammatory cytokines such as TNF-α and IL-6, which, in turn, induces chronic inflammation and increases susceptibility to cancer and loss of Treg cell function (25). Indeed, miR-146a suppresses the growth and migration and induces apoptosis of NSCLC cells (26). miR-146a also induces G0/G1 cell cycle, which may suppress the proliferation of lung cancer cells (27). Interestingly, miR-146a expression is significantly lower in lung cancer tissue, which suggests that it acts as a tumor suppressor *via* targeting EGFR expression (26). EGFR-tyrosine kinase inhibitors (EGFR-TKIs) are used successfully as targeted therapies in lungs cancer (26, 28).

High levels of miR-146a-5p are seen in the serum and tissue of NSCLC patients (29). miRNA146a-5P has effects on the survival and proliferation of NSCLC cells through binding and suppression of TRAF-6 (29). Interestingly, the expression of serum miR-146a, but not miR-155-5p, is increased in patients progressing from stage I to stage II NSCLC (30). Indeed, elevated miR-146a levels in serum exosomes have been proposed as a diagnostic marker in the early stages of NSCLC (30). However, poor prognosis has shown related to the lower serum levels and tissue expression of miR146a (31). In the current study we did not find any significant changes in the expression of miR-146 or miR-155 in type and stages of NSCLC patients, indicating that progression through disease stages did not affect the expression of these miRNAs.

miR-155 expression is increased in the lung tissue of NSCLC patients and correlates with disease progression (32). miR-155 increases the survival of Treg cells by increasing sensitivity of these cells to IL-2 *via* attenuating suppressor of cytokine signaling 1 (SOCS1) pathways (15). Moreover, miR-155 positively feedbacks on NF-κB signaling by inhibiting SH-2 containing inositol 5’ polyphosphatase 1 (SHIP-1) and SOCS1 (33). miR-155 also acts as a positive regulator of cytokine production in macrophages (34). Upregulation of miR-155 expression in lung cancer tissues, plasma, and sputum is associated with an increased risk of NSCLC where there are no current good diagnostic markers (35–37). Moreover, miR-125a-3p, miR-125b-5p, miR-155-5p are reported to be increased in stage I of lung adenocarcinoma (38). In the current study, no significant difference in the expression of miR-155 in the PBMC of NSCLC and healthy controls was seen, although miR-155 is significantly upregulated in several NSCLC cell lines (SPC-A-1, A549, H2170) (39).

We show that CD3/CTLA4 frequency was markedly higher in NSCLC patients and that this positively correlated with miR146a in patients with a CC genotype. CTLA-4 is a T cell-restricted immune checkpoint that inhibits the T-cell response when it attaches to B7 on antigen-presenting cells (APCs). Moreover, CTLA-4 suppresses IL-2 production and thereby blocks cell-cycle progression, leading to induction and maintenance of T-cell tolerance. Under physiological conditions, CTLA-4 decreased the T-cell response to foreign antigens as well as to autoantigens. T-cell expression of CTLA-4 is elevated by TGF-β, a suppressive cytokine secreted by the tumor cells (40–42). We report here a negative correlation of miR-146 expression with TGF-β. This suggests that using miR-146a may be able to be used as a prediction of the clinical response of advanced NSCLC patients to immune checkpoint inhibitors (ICI), and this may be considered a limitation of the study and should be investigated in future studies. miR-146 tightly regulates cytokines such as TNF-α and IL-1β through different signaling pathways including NF-κB and MEK-1/2 and JNK-1/2 (43). miR-146a has a major effect on programmed cell death, and its overexpression suppresses cell migration and proliferation in NSCLC cell lines and has potential as a tumor-suppressive and anti-inflammatory agent. In addition, mir-155 is involved in the crosstalk between cancer and inflammation and additional research in this (44).

TGF-β is linked with cancer progression and is associated with poor prognosis of NSCLC patients (45, 46). We show elevated serum TGF-β levels in NSCLC patients that negatively correlated with the expression of miR-146a in PBMC. Interestingly, patients with high serum levels of miR-146a achieved a higher overall response rate to therapies and a longer survival time (31).

IL-6 plays an important role in early stages of lung cancer and potentiates immune responses resulting in cell proliferation and expansion of the tumor mass (47). Higher serum IL-6 levels in NSCLC patients in the current study was not correlated with miR-146a or miR-155 expression, although a positive correlation between miR-146a and IL-1β was observed especially in patients with a GG genotype. miR-146a regulates IL-1β expression (48–50), and IL-1β plays an important role in tumor progression by enhancing angiogenesis, amplifying myeloid-derived suppressive cells (MDSCs) and shifting macrophages towards an M2 phenotype (51, 52).

miR-146a and miR-155 are expressed in Treg cells (53), and there was a negative correlation between miR-146a and the frequency of Treg cells in NSCLC patients with a GG genotype. Furthermore, there was a positive correlation between miR-155 and CTLA4+ T-cell frequency in patients with an AT genotype. Overall, these changes (low miR146 with high Treg and high CD3/CTLA-4) may suggest a damping of the immune system response with poor disease prognosis. Among the 33 NSCLC patients, only eight patients had a CC genotype, and these had a positive correlation between miR-146a expression and the frequency of CD3+CTLA4+ lymphocytes.

In conclusion, the current study shows a connection between downregulation of miR-146 with increased serum levels of TGF-β.Thus, blocking TGF-β using monoclonal antibodies may potentiate the effects of ICIs such as CTLA4 blockers, resulting in an immune brake, enhancing T-cell cytotoxicity and enhancing cancer cell killing. The data presented here have clinical implications for NSCLC; however, measuring miR-146 in parallel with serum cytokine levels may provide a better evaluation of the immune response and outcome of disease.

## Data Availability Statement

The raw data supporting the conclusions of this article will be made available by the authors, without undue reservation.

## Ethics Statement

The studies involving human participants were reviewed and approved by the National Research Institute Tuberculosis and Lung Disease. The patients/participants provided their written informed consent to participate in this study.

## Author Contributions

ND and EM did experiments. ND and NR did statistical analysis. SS and BS as oncologists helped in patient’s approval and confirmed patient’s status. ND and EM wrote the first and final draft of the paper. SDA revised the paper and comments for discussion. IMA as a native writer edited the article and advised last edition. All authors contributed to the article and approved the submitted version.

## Funding

EM is funded by the Iran National Science Foundation (INSF), grant number 98003666.

## Conflict of Interests

The authors declare that the research was conducted in the absence of any commercial or financial relationships that could be construed as a potential conflict of interest.

## Publisher’s Note

All claims expressed in this article are solely those of the authors and do not necessarily represent those of their affiliated organizations, or those of the publisher, the editors and the reviewers. Any product that may be evaluated in this article, or claim that may be made by its manufacturer, is not guaranteed or endorsed by the publisher.

## References

[1] Siegel R Naishadham D Jemal A. Cancer Statistics, 2013. CA Cancer J Clin (2013) 63(1):11–30. doi: 10.3322/caac.21166 23335087

[2] Hou J Meng F Chan LW Cho WC Wong SC. Circulating Plasma MicroRNAs As Diagnostic Markers for NSCLC. Front Genet (2016) 7:193. doi: 10.3389/fgene.2016.00193 27857721 PMC5093122

[3] Naeli P Yousefi F Ghasemi Y Savardashtaki A Mirzaei H. The Role of MicroRNAs in Lung Cancer: Implications for Diagnosis and Therapy. Curr Mol Med (2020) 20(2):90–101. doi: 10.2174/1566524019666191001113511 31573883

[4] Rotte A. Combination of CTLA-4 and PD-1 Blockers for Treatment of Cancer. J Exp Clin Cancer Res: CR (2019) 38(1):255. doi: 10.1186/s13046-019-1259-z 31196207 PMC6567914

[5] Buchbinder EI Desai A. CTLA-4 and PD-1 Pathways. Am J Clin Oncol (2016) 39(1):98–106. doi: 10.1097/coc.0000000000000239 26558876 PMC4892769

[6] Maemondo M Inoue A Kobayashi K Sugawara S Oizumi S Isobe H. Gefitinib or Chemotherapy for Non–Small-Cell Lung Cancer With Mutated EGFR. N Engl J Med (2010) 362(25):2380–8. doi: 10.1056/NEJMoa0909530 20573926

[7] Gainor JF Varghese AM Ou SH Kabraji S Awad MM Katayama R. ALK Rearrangements Are Mutually Exclusive With Mutations in EGFR or KRAS: An Analysis of 1,683 Patients With Non-Small Cell Lung Cancer. Clin Cancer Res (2013) 19(15):4273–81. doi: 10.1158/1078-0432.CCR-13-0318 PMC3874127 23729361

[8] Zhou C Wu Y-L Chen G Feng J Liu X Wang C. Erlotinib Versus Chemotherapy as First-Line Treatment for Patients With Advanced EGFR Mutation-Positive Non-Small-Cell Lung Cancer (OPTIMAL, CTONG-0802): A Multicentre, Open-Label, Randomised, Phase 3 Study. Lancet Oncol (2011) 12(8):735–42. doi: 10.1016/s1470-2045(11)70184-x 21783417

[9] Howlader N Forjaz G Mooradian MJ Meza R Kong CY Cronin KA. The Effect of Advances in Lung-Cancer Treatment on Population Mortality. N Engl J Med (2020) 383(7):640–9. doi: 10.1056/NEJMoa1916623 PMC8577315 32786189

[10] Kitamura T Qian B-Z Pollard JW. Immune Cell Promotion of Metastasis. Nat Rev Immunol (2015) 15(2):73–86. doi: 10.1038/nri3789 25614318 PMC4470277

[11] Piotrowski I Kulcenty K Suchorska W. Interplay Between Inflammation and Cancer. Rep Pract Oncol Radiother (2020) 25(3):422–7. doi: 10.1016/j.rpor.2020.04.004 PMC7191124 32372882

[12] Yi M Xu L Jiao Y Luo S Li A Wu K. The Role of Cancer-Derived microRNAs in Cancer Immune Escape. J Hematol Oncol (2020) 13(1):25. doi: 10.1186/s13045-020-00848-8 32222150 PMC7103070

[13] Paladini L Fabris L Bottai G Raschioni C Calin GA Santarpia L. Targeting microRNAs as Key Modulators of Tumor Immune Response. J Exp Clin Cancer Res: CR (2016) 35:103. doi: 10.1186/s13046-016-0375-2 27349385 PMC4924278

[14] Liu G Abraham E. MicroRNAs in Immune Response and Macrophage Polarization. Arterioscler Thromb Vasc Biol (2013) 33(2):170–7. doi: 10.1161/ATVBAHA.112.300068 PMC3549532 23325473

[15] Testa U Pelosi E Castelli G Labbaye C. miR-146 and miR-155: Two Key Modulators of Immune Response and Tumor Development. Non-Coding RNA (2017) 3(3):22. doi: 10.3390/ncrna3030022 PMC5831915 29657293

[16] Dezfuli NK Adcock IM Alipoor SD Seyfi S Salimi B Golchin MM. The miR-146a SNP Rs2910164 and miR-155 SNP Rs767649 Are Risk Factors for Non-Small Cell Lung Cancer in the Iranian Population. Can Respir J (2020) 2020:1–8. doi: 10.1155/2020/8179415 PMC7700047 33294082

[17] Zhao Z Qi F Liu T Fu W. Effect of miR-146a and miR-155 on Cardiac Xenotransplantation. Exp Ther Med (2016) 12(6):3972–8. doi: 10.3892/etm.2016.3867 PMC5228279 28101175

[18] Huffaker TB Hu R Runtsch MC Bake E Chen X Zhao J. Epistasis Between microRNAs 155 and 146a During T Cell-Mediated Antitumor Immunity. Cell Rep (2012) 2(6):1697–709. doi: 10.1016/j.celrep.2012.10.025 PMC3628775 23200854

[19] Fortunato O Boeri M Verri C Moro M Sozzi G. Therapeutic Use of microRNAs in Lung Cancer. BioMed Res Int (2014) 2014:756975. doi: 10.1155/2014/756975 25309923 PMC4182304

[20] Mortaz E Redegeld FA Nijkamp FP Engels F. Dual Effects of Acetylsalicylic Acid on Mast Cell Degranulation, Expression of Cyclooxygenase-2 and Release of Pro-Inflammatory Cytokines. Biochem Pharmacol (2005) 69(7):1049–57. doi: 10.1016/j.bcp.2004.12.018 15763541

[21] He H Jazdzewski K Li W Liyanarachchi S Nagy R Volinia S. The Role of microRNA Genes in Papillary Thyroid Carcinoma. Proc Natl Acad Sci USA (2005) 102(52):19075–80. doi: 10.1073/pnas.0509603102 PMC1323209 16365291

[22] Lu LF Boldin MP Chaudhry A Lin LL Taganov KD Hanada T. Function of miR-146a in Controlling Treg Cell-Mediated Regulation of Th1 Responses. Cell (2010) 142(6):914–29. doi: 10.1016/j.cell.2010.08.012 PMC3049116 20850013

[23] Wang H Li X Li T Wang L Wu X Liu J. Multiple Roles of microRNA-146a in Immune Responses and Hepatocellular Carcinoma. Oncol Lett (2019) 18(5):5033–42. doi: 10.3892/ol.2019.10862 PMC6781720 31612014

[24] Labbaye C Testa U. The Emerging Role of MIR-146A in the Control of Hematopoiesis, Immune Function and Cancer. J Hematol Oncol (2012) 5:13. doi: 10.1186/1756-8722-5-13 22453030 PMC3342163

[25] Eide HA Halvorsen AR Sandhu V Fåne A Berg J Haakensen VD. Non-Small Cell Lung Cancer Is Characterised by a Distinct Inflammatory Signature in Serum Compared With Chronic Obstructive Pulmonary Disease. Clin Trans Immunol (2016) 5(11):e109. doi: 10.1038/cti.2016.65 PMC5133367 27990285

[26] Chen G Umelo IA Lv S Teugels E Fostier K Kronenberger P. miR-146a Inhibits Cell Growth, Cell Migration and Induces Apoptosis in Non-Small Cell Lung Cancer Cells. PLoS One (2013) 8(3):e60317. doi: 10.1371/journal.pone.0060317 23555954 PMC3608584

[27] Li YL Wang J Zhang CY Shen YQ Wang HM Ding L. MiR-146a-5p Inhibits Cell Proliferation and Cell Cycle Progression in NSCLC Cell Lines by Targeting CCND1 and CCND2. Oncotarget (2016) 7(37):59287–98. doi: 10.18632/oncotarget.11040 PMC5312312 27494902

[28] Lu J Zhan Y Feng J Luo J Fan S. MicroRNAs Associated With Therapy of Non-Small Cell Lung Cancer. Int J Biol Sci (2018) 14(4):390–7. doi: 10.7150/ijbs.22243 PMC5930471 29725260

[29] Liu X Liu B Li R Wang F Wang N Zhang M. miR-146a-5p Plays an Oncogenic Role in NSCLC *via* Suppression of TRAF6. Front Cell Dev Biol (2020) 8:847. doi: 10.3389/fcell.2020.00847 33015045 PMC7493784

[30] Wu Q Yu L Lin X Zheng Q Zhang S Chen D. Combination of Serum miRNAs With Serum Exosomal miRNAs in Early Diagnosis for Non-Small-Cell Lung Cancer. Cancer Manage Res (2020) 12:485–95. doi: 10.2147/CMAR.S232383 PMC6982436 32021461

[31] Wu C Cao Y He Z He J Hu C Duan H. Serum Levels of miR-19b and miR-146a as Prognostic Biomarkers for Non-Small Cell Lung Cancer. Tohoku J Exp Med (2014) 232(2):85–95. doi: 10.1620/tjem.232.85 24531034

[32] Xue X Liu Y Wang Y Meng M Wang K Zang X. MiR-21 and MiR-155 Promote non-Small Cell Lung Cancer Progression by Downregulating SOCS1, SOCS6, and PTEN. Oncotarget (2016) 7(51):84508–19. doi: 10.18632/oncotarget.13022 PMC5356677 27811366

[33] Mehta A Baltimore D. MicroRNAs as Regulatory Elements in Immune System Logic. Nat Rev Immunol (2016) 16(5):279–94. doi: 10.1038/nri.2016.40 27121651

[34] Kurowska-Stolarska M Alivernini S Ballantine LE Asquith DL Millar NL Gilchrist DS. MicroRNA-155 as a Proinflammatory Regulator in Clinical and Experimental Arthritis. Proc Natl Acad Sci (2011) 108(27):11193–8. doi: 10.1073/pnas.1019536108 PMC3131377 21690378

[35] Xie K Ma H Liang C Wang C Qin N Shen W. A Functional Variant in miR-155 Regulation Region Contributes to Lung Cancer Risk and Survival. Oncotarget (2015) 6(40):42781–92. doi: 10.18632/oncotarget.5840 PMC4767470 26543233

[36] Lamichhane SR Thachil T De Ieso P Gee H Moss SA Milic N. Prognostic Role of MicroRNAs in Human Non-Small-Cell Lung Cancer: A Systematic Review and Meta-Analysis. Dis Markers (2018) 2018:1–17. doi: 10.1155/2018/8309015 PMC6260404 30538784

[37] Papadaki C Monastirioti A Rounis K Makrakis D Kalbakis K Nikolaou C. Circulating MicroRNAs Regulating DNA Damage Response and Responsiveness to Cisplatin in the Prognosis of Patients With Non-Small Cell Lung Cancer Treated With First-Line Platinum Chemotherapy. Cancers (Basel) (2020) 12(5):1–21. doi: 10.3390/cancers12051282 PMC7281609 32438598

[38] Zeybek A Oz N Kalemci S Edgünlü T Kızıltuğ MT Tosun K. Diagnostic Value of MiR-125b as a Potential Biomarker for Stage I Lung Adenocarcinoma. Curr Mol Med (2019) 19(3):216–27. doi: 10.2174/1566524019666190314113800 30868951

[39] Liu F Song D Wu Y Liu X Zhu J Tang Y. MiR-155 Inhibits Proliferation and Invasion by Directly Targeting PDCD4 in Non-Small Cell Lung Cancer. Thorac Cancer (2017) 8(6):613–9. doi: 10.1111/1759-7714.12492 PMC5668490 28842954

[40] Hodi FS O’Day SJ McDermott DF Weber RW Sosman JA Haanen JB. Improved Survival With Ipilimumab in Patients With Metastatic Melanoma. N Engl J Med (2010) 363(8):711–23. doi: 10.1056/NEJMoa1003466 PMC3549297 20525992

[41] Frey AB Monu N. Signaling Defects in Anti-Tumor T Cells. Immunol Rev (2008) 222:192–205. doi: 10.1111/j.1600-065X.2008.00606.x 18364003 PMC3731145

[42] Herzberg B Campo MJ Gainor JF. Immune Checkpoint Inhibitors in Non-Small Cell Lung Cancer. Oncologist (2017) 22(1):81–8. doi: 10.1634/theoncologist.2016-0189 PMC5313266 27534574

[43] Tahamtan A Teymoori-Rad M Nakstad B Salimi V. Anti-Inflammatory MicroRNAs and Their Potential for Inflammatory Diseases Treatment. Front Immunol (2018) 9:1377. doi: 10.3389/fimmu.2018.01377 29988529 PMC6026627

[44] Tili E Croce CM Michaille JJ. miR-155: On the Crosstalk Between Inflammation and Cancer. Int Rev Immunol (2009) 28(5):264–84. doi: 10.1080/08830180903093796 19811312

[45] Li J Shen C Wang X Lai Y Zhou K Li P. Prognostic Value of TGF-Beta in Lung Cancer: Systematic Review and Meta-Analysis. BMC Cancer (2019) 19(1):691. doi: 10.1186/s12885-019-5917-5 31307405 PMC6631541

[46] Domagala-Kulawik J. The Role of the Immune System in Non-Small Cell Lung Carcinoma and Potential for Therapeutic Intervention. Transl Lung Cancer Res (2015) 4(2):177–90. doi: 10.3978/j.issn.2218-6751.2015.01.11 PMC4384216 25870800

[47] Qu Z Sun F Zhou J Li L Shapiro SD Xiao G. Interleukin-6 Prevents the Initiation But Enhances the Progression of Lung Cancer. Cancer Res (2015) 75(16):3209–15. doi: 10.1158/0008-5472.CAN-14-3042 PMC4537823 26122841

[48] Perry MM Moschos SA Williams AE Shepherd NJ Larner-Svensson HM Lindsay MA. Rapid Changes in microRNA-146a Expression Negatively Regulate the IL-1beta-Induced Inflammatory Response in Human Lung Alveolar Epithelial Cells. J Immunol (2008) 180(8):5689–98. doi: 10.4049/jimmunol.180.8.5689 PMC2639646 18390754

[49] Jiang S Hu Y Deng S Deng J Yu X Huang G. miR-146a Regulates Inflammatory Cytokine Production in Porphyromonas Gingivalis Lipopolysaccharide-Stimulated B Cells by Targeting IRAK1 But Not TRAF6. Biochim Biophys Acta Mol Basis Dis (2018) 1864(3):925–33. doi: 10.1016/j.bbadis.2017.12.035 PMC5803357 29288795

[50] Nahid MA Satoh M Chan EK. Interleukin 1beta-Responsive MicroRNA-146a Is Critical for the Cytokine-Induced Tolerance and Cross-Tolerance to Toll-Like Receptor Ligands. J Innate Immun (2015) 7(4):428–40. doi: 10.1159/000371517 PMC4485520 25896300

[51] Castro D Moreira M Gouveia AM Pozza DH De Mello RA. MicroRNAs in Lung Cancer. Oncotarget (2017) 8(46):81679–85. doi: 10.18632/oncotarget.20955 PMC5655318 29113423

[52] Alivernini S Gremese E McSharry C Tolusso B Ferraccioli G McInnes IB. MicroRNA-155-at the Critical Interface of Innate and Adaptive Immunity in Arthritis. Front Immunol (2017) 8:1932. doi: 10.3389/fimmu.2017.01932 29354135 PMC5760508

[53] Liu C Li N Liu G. The Role of MicroRNAs in Regulatory T Cells. J Immunol Res (2020) 2020:3232061. doi: 10.1155/2020/3232061 32322593 PMC7154970

