# Supplementary material for: Evaluation Expression of miR-146a and miR-155 in Non-Small-Cell Lung Cancer Patients
Source: Front Oncol. 2021 Nov 1;11:715677. doi: 10.3389/fonc.2021.715677 (PMC8591170; doi:10.3389/fonc.2021.715677)
Supplement: Supplementary file 1 [file Table_1.doc]

**Supplementary file: Table 1:** RNA and DNA concentrations isolated from healthy subjects peripheral blood cells measure by  **Nonodrop 2000**

|  | RNA | | | DNA | | |
| --- | --- | --- | --- | --- | --- | --- |
| Control subjects | Concentration  (ng/μl) | 260/280  (nm) | 260/230  (nm) | Concentration  (ng/μl) | 260/280  (nm) | 260/230  (nm) |
| 1 | 155.5 | 1.91 | 1.49 | 42.0 | 1.91 | 1.78 |
| 2 | 46 | 1.2 | 1.13 | 101 | 1.8 | 1.25 |
| 3 | 13.1 | 1.93 | 0.17 | 107 | 1.82 | 1.31 |
| 4 | 90.8 | 1.59 | 0.92 | 121 | 1.84 | 1.3 |
| 5 | 69.8 | 1.8 | 1.01 | 115 | 1.84 | 1.5 |
| 6 | 100.3 | 1.7 | 1.17 | 105 | 1.83 | 1.36 |
| 7 | 293.3 | 1.72 | 0.51 | 144 | 1.87 | 1.36 |
| 8 | 143.2 | 1.94 | 1.45 | 113.1 | 1.78 | 1.22 |
| 9 | 46.3 | 1.4 | 0.81 | 327 | 1.87 | 1.41 |
| 10 | 291.1 | 1.7 | 1.51 | 210 | 1.78 | 2.21 |
| 11 | 339.0 | 1.7 | 0.7 | 165 | 1.81 | 1.16 |
| 12 | 150 | 1.7 | 0.92 | 25.5 | 1.71 | 0.91 |
| 13 | 158 | 1.9 | 1.4 | 10 | 1.81 | 1.25 |
| 14 | 111.5 | 1.2 | 0.89 | 18.7 | 1.85 | 1.4 |
| 15 | 95.5 | 1.4 | 1.2 | 19.7 | 1.64 | 1.29 |
| 16 | 118 | 1.1 | 0.99 | 39.5 | 1.8 | 1.42 |
| 17 | 33.7 | 1.71 | 0.96 | 24.8 | 1.88 | 1.25 |
| 18 | 174 | 2.1 | 1.4 | 78.8 | 1.84 | 1.32 |
| 19 | 120 | 1.52 | 0.87 | 113.1 | 1.77 | 1.79 |
| 20 | 33 | 1.41 | 0.64 | 211 | 1.8 | 1.2 |
| 21 | 122.8 | 1.91 | 1.82 | 44.2 | 1.5 | 0.87 |
| 22 | 341.8 | 1.78 | 0.57 | 75.1 | 1.3 | 1.41 |
| 23 | 184.5 | 1.65 | 0.31 | 115.1 | 1.4 | 1.12 |
| 24 | 12.1 | 1.84 | 0.24 | 87.1 | 1.74 | 1.61 |
| 25 | 91.2 | 1.62 | 0.38 | 111.2 | 1.65 | 1.431 |
| 26 | 31.2 | 1.51 | 0.29 | 77.1 | 1.33 | 1.2 |
| 27 | 115.2 | 1.54 | 0.79 | 114.3 | 1.22 | 0.97 |
| 28 | 21.4 | 1.98 | 0.32 | 51.5 | 1.38 | 1.11 |
| 29 | 21.9 | 2 | 0.31 | 78.1 | 1.61 | 0.89 |
| 30 | 67.4 | 1.91 | 1.01 | 91.2 | 1.87 | 1.54 |

**Table 2: RNA and DNA concentrations isolated from NSCLC patients peripheral blood cells measure by Nanodrop-2000**

|  | RNA | | | DNA | | |
| --- | --- | --- | --- | --- | --- | --- |
| NSCLC Patients | Concentration  (ng/μl) | 260/280  (nm) | 260/230  (nm) | Concentration  (ng/μl) | 260/280  (nm) | 260/230  (nm) |
| 1 | 117.6 | 1.84 | 2.03 | 25.6 | 1.98 | 1.57 |
| 2 | 31.9 | 1.72 | 1.28 | 71.5 | 1.27 | 1.16 |
| 3 | 33.8 | 1.78 | 1.18 | 115.1 | 1.3 | 1.1 |
| 4 | 106 | 1.83 | 1.77 | 211.1 | 1.66 | 1.43 |
| 5 | 109.1 | 1.85 | 1.55 | 384.7 | 1.57 | 1.20 |
| 6 | 64.1 | 1.73 | 0.32 | 56.1 | 1.38 | 0.98 |
| 7 | 56.0 | 1.83 | 1.2 | 117.4 | 1.33 | 1.2 |
| 8 | 84.8 | 1.81 | 0.91 | 364 | 1.88 | 1.3 |
| 9 | 95.6 | 1.97 | 0.91 | 88.3 | 1.43 | 0.89 |
| 10 | 85.9 | 1.96 | 1.02 | 102.1 | 1.61 | 1.34 |
| 11 | 57.4 | 1.8 | 1.1 | 114.3 | 1.7 | 1.21 |
| 12 | 101.2 | 1.32 | 0.94 | 79.1 | 1.4 | 0.98 |
| 13 | 43.1 | 1.61 | 0.99 | 55.1 | 1.1 | 0.87 |
| 14 | 101.1 | 1.6 | 1.1 | 11.7 | 2.02 | 1.51 |
| 15 | 73.2 | 1.32 | 1.2 | 81.4 | 1.8 | 1.2 |
| 16 | 122 | 1.7 | 1.31 | 32.5 | 0.62 | 1.11 |
| 17 | 53.2 | 1.5 | 0.88 | 106.1 | 1.7 | 1.41 |
| 18 | 96.1 | 1.7 | 0.45 | 58.7 | 1.48 | 1.09 |
| 19 | 243.1 | 1.5 | 0.99 | 118.6 | 1.74 | 1.2 |
| 20 | 79.6 | 1.2 | 1.5 | 69.1 | 1.9 | 1.6 |
| 21 | 81.4 | 1.5 | 1.1 | 98.1 | 1.8 | 1.3 |
| 22 | 133 | 1.96 | 1.03 | 113 | 1.6 | 1.3 |
| 23 | 105 | 1.80 | 1.3 | 21.1 | 1.1 | 0.81 |
| 24 | 47 | 1.61 | 0.54 | 91.1 | 1.71 | 1.5 |
| 25 | 23.1 | 1.4 | 1.45 | 45.9 | 1.4 | 1.1 |
| 26 | 106.1 | 1.9 | 0.96 | 102.1 | 1.81 | 1.3 |
| 27 | 79.6 | 1.48 | 0.79 | 33 | 1.6 | 1.1 |
| 28 | 33.1 | 1.8 | 0.67 | 103.1 | 1.7 | 1.51 |
| 29 | 101.8 | 1.7 | 1.18 | 109.3 | 1.68 | 1.2 |
| 30 | 21.5 | 1.3 | 0.89 | 59.2 | 1.8 | 1.2 |
| 31 | 66.4 | 1.54 | 0.56 | 118.1 | 1.5 | 1.34 |
| 32 | 112.7 | 1.6 | 0.86 | 85.1 | 1.7 | 1.31 |
| 33 | 278.1 | 1.57 | 1.09 | 101.2 | 1.9 | 1.61 |
